# In-Cell NMR Study of Tau and MARK2 Phosphorylated Tau

**DOI:** 10.3390/ijms20010090

**Published:** 2018-12-26

**Authors:** Shengnan Zhang, Chuchu Wang, Jinxia Lu, Xiaojuan Ma, Zhenying Liu, Dan Li, Zhijun Liu, Cong Liu

**Affiliations:** 1 Interdisciplinary Research Center on Biology and Chemistry, Shanghai Institute of Organic Chemistry, Chinese Academy of Sciences, 26 Qiuyue Road, Shanghai 201210, China; wangcc@sioc.ac.cn (C.W.); maxiaojuan@sioc.ac.cn (X.M.); liuzy@sioc.ac.cn (Z.L.); 2 Interdisciplinary Research Center on Biology and Chemistry, Shanghai Institute of Organic Chemistry, University of the Chinese Academy of Sciences, 19 A Yuquan Road, Shijingshan District, Beijing 100049, China; 3 Key Laboratory for the Genetics of Developmental and Neuropsychiatric Disorders (Ministry of Education), Bio-X Institutes, Shanghai Jiao Tong University, Shanghai 200030, China; lujx@sjtu.edu.cn (J.L.); lidan2017@sjtu.edu.cn (D.L.); 4 National Facility for Protein Science in Shanghai, Zhangjiang Lab, Shanghai Advanced Research Institute, Chinese Academy of Sciences, Shanghai 201210, China; liuzhijun@sari.ac.cn

**Keywords:** in-cell NMR, Tau, MARK2 phosphorylation, mammalian cells

## Abstract

The intrinsically disordered protein, Tau, is abundant in neurons and contributes to the regulation of the microtubule (MT) and actin network, while its intracellular abnormal aggregation is closely associated with Alzheimer’s disease. Here, using in-cell Nuclear Magnetic Resonance (NMR) spectroscopy, we investigated the conformations of two different isoforms of Tau, Tau40 and k19, in mammalian cells. Combined with immunofluorescence imaging and western blot analyses, we found that the isotope-enriched Tau, which was delivered into the cultured mammalian cells by electroporation, is partially colocalized with MT and actin filaments (F-actin). We acquired the NMR spectrum of Tau in human embryonic kidney 293 (HEK-293T) cells, and compared it with the NMR spectra of Tau added with MT, F-actin, and a variety of crowding agents, respectively. We found that the NMR spectrum of Tau in complex with MT best recapitulates the in-cell NMR spectrum of Tau, suggesting that Tau predominantly binds to MT at its MT-binding repeats in HEK-293T cells. Moreover, we found that disease-associated phosphorylation of Tau was immediately eliminated once phosphorylated Tau was delivered into HEK-293T cells, implying a potential cellular protection mechanism under stressful conditions. Collectively, the results of our study reveal that Tau utilizes its MT-binding repeats to bind MT in mammalian cells and highlight the potential of using in-cell NMR to study protein structures at the residue level in mammalian cells.

## 1. Introduction

The intracellular environment is highly crowded, viscous, and packed with different proteins, nuclear acids, lipids, and other biomolecules. The structure and function of a certain protein is defined by the interplay between the protein and its neighboring biomolecules in the intracellular environment [1,2,3,4]. Therefore, it is of great importance to study the atomic structure and conformational dynamics of proteins within living cells, especially for intrinsically disordered proteins that lack well-folded 3D structures in the absence of binding partners [5,6,7]. However, conventional structural biology methods—including X-ray crystallography, solution NMR, and Cryogenic Electron Microscopy (cryo-EM)—deal with proteins purified in vitro, either in an aqueous solution or in a crystal structure, where the environment is highly simplified and fundamentally distinct from that inside the cell. Notably, recently developed in-cell NMR spectroscopy has emerged as a powerful technique to characterize the structures of proteins of interest within living cells at an atomic level [2,3,8,9,10,11]. To date, in-cell NMR has been successfully developed to characterize the protein structure [12], dynamics [13], and interactions [14,15] in bacteria. However, in contrast to eukaryotic cells, prokaryotic cells only reveal limited biological activities and lack significant biological processes such as protein maturation and post-translational modification, which can alter the structure and function of proteins. Thus, much effort has been devoted to extending in-cell NMR studies in eukaryotic cells, where the most technically challenging work is in introducing selective isotope-labeled proteins of interest in cells for NMR detection. Microinjection into *Xenopus laevis* oocytes [16,17,18], endocytotic transportation mediated by a cell-penetrating peptide [1,19], and diffusion through pore-forming toxins [20] have already been developed to successfully deliver isotopically labeled proteins purified in vitro to eukaryotic cells. Most recently, electroporation was shown to be as an effective and general approach to deliver isotope-labeled proteins into different types of mammalian cells [6,21]. Therefore, advances in the methodology of in-cell NMR pave the way toward investigating the structures and conformational dynamics of different proteins in the intracellular environment. 

Tau is a typical intrinsically disordered protein that is highly abundant in the central nervous system [22,23]. It is capable of binding to a variety of proteins and other biomolecules including MT, heparin, and lipid molecules [24,25,26,27,28]. The physiological function of Tau is involved in the regulation and stabilization of the MT and actin network [29,30,31]. Tau contains multiple sites for post-translational modifications (e.g., phosphorylation, acetylation, methylation, and ubiquitination) under different cellular conditions for either the regulation of its normal function or in the pathogenesis of a disease [32]. For instance, hyperphosphorylation of Tau leads to the detachment of Tau from MT into the cytosol and the formation of abnormal filamentous amyloid aggregates [33,34,35]. These filamentous aggregates are the pathological hallmarks of a variety of neurodegenerative diseases including Alzheimer’s disease (AD) [36], Pick’s disease [37], and progressive supranuclear palsy [38]. Human tau in the brain has six isoforms that range from 352 to 441 amino acids in length [39]. The six isoforms differ in the number of MT-binding repeats (three or four) and insertions in the N-terminal projection domain (zero, one, or two). Cryo-EM studies have revealed that the MT-binding repeats are composed of an amyloid fibril core of filamentous Tau aggregates isolated from patient brains [36,37]. In contrast to the intensive investigation on the aggregated forms of Tau formed under pathogenic conditions, the structural studies on the soluble form of Tau—especially the conformation of Tau in the intracellular environment, and its relationship with its physiological function—are very limited. 

In this study, we investigated the structures of two different isoforms of Tau, Tau40 and k19, in mammalian cells using in-cell NMR spectroscopy. The isotopically labeled Tau proteins were efficiently delivered into HEK-293T cells by electroporation. In combination with immunofluorescence imaging and in vitro NMR titration experiments, we confirmed that Tau/k19 can bind to both MT and F-actin in vitro, and they partially colocalize with MT and F-actin in the mammalian cells. The solution NMR spectrum of k19 in complex with MT best recapitulates the in-cell NMR spectrum of k19, suggesting that k19 predominantly binds to MT in the HEK-293T cells. Moreover, we found that microtubule affinity-regulating kinase 2 (MARK2) phosphorylated k19 was immediately dephosphorylated once being delivered into the HEK-293T cells. Our study reveals that Tau utilizes its MT-binding repeats to bind MT in mammalian cells, and highlights the potential of using in-cell NMR to study protein structure at the residue level in mammalian cells. 

## 2. Results

### 2.1. In-Cell NMR Study of Tau k19

**Figure 1:**
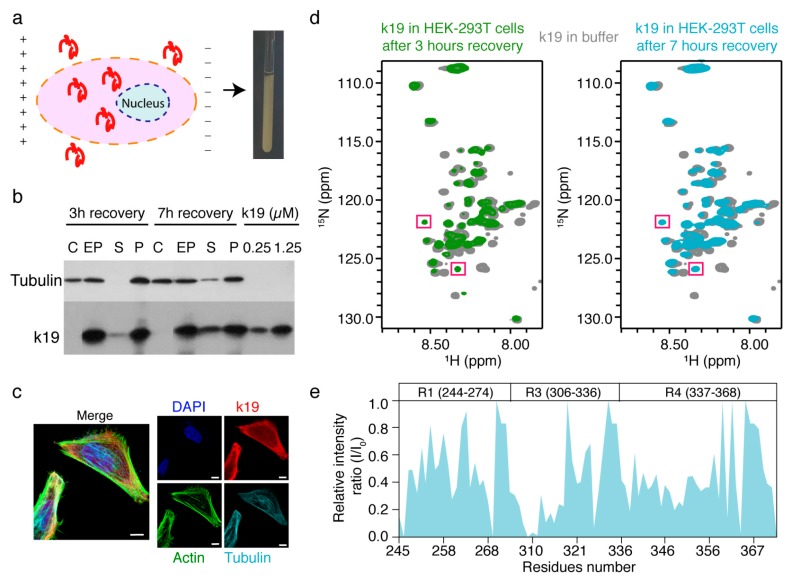
Characterization of the electroporated Tau k19 in mammalian cells. (**a**) Scheme of the electroporation procedure to deliver isotope-labeled k19 into mammalian cells and the resulting in-cell NMR sample. The orange and blue dotted circle stand for the cell and nucleus memberane of mammalian cells, respectively. + and − represent the electrical property of the electrode; (**b**) Western blot analysis of k19 electroporated into HEK-293T cells. Electroporated cells (EP) and untreated control cells (C) were diluted 10 times to be loaded with the same volume of purified k19 with indicated concentrations. S and P stand for the supernatant medium and cell pellet, respectively, of the in-cell NMR sample after NMR data collection; (**c**) Immunofluorescence detection of delivered k19 in SH-SY5Y cells after 7 h recovery. DAPI is used to stain nucleus. Scale bar, 7.5 μm; (**d**) Overlay of 2D in-cell NMR spectra of k19 in HEK-293T cells with 3 (green) and 7 (blue) hours recovery and in buffer (grey), respectively. The two additional crosspeaks in the in-cell NMR spectra are highlighted by the magenta boxes; (**e**) Residue-specific relative intensity ratio (I/I_0_) of k19 in HEK-293T cells with 7 h recovery to that of k19 in buffer. Domain organization of k19 is illustrated on the top of the graph.

We first sought to investigate the structure of the three MT-binding repeats of Tau–k19 in mammalian cells using in-cell NMR, since k19 with 98 residues is much easier to study by NMR compared to Tau40 with 441 residues. Moreover, k19 contains the major AD related phosphorylation sites, and consists of the core sequence of filamentous Tau aggregates that is highly related to the pathology of Tau to AD. ^15^N-labeled k19 was overexpressed and purified from *Escherichia coli (E. coli)*, and was then delivered into the HEK-293T cells by using a recently developed electroporation-based protocol (Figure 1a). To ensure that k19 is effectively delivered into the cells, we optimized different variables in the protocol. We optimized the concentration of k19 incubated with cells for electroporation ranging from 50–500 μM. We also optimized the electroporation parameters including pulse voltage (1200, 1300, or 1400 V), pulse width (10 or 20 milliseconds (ms)), and number of pulses (1 or 2). Finally, with a pulse program of 1400 V, 20 milliseconds (ms) and 1 pulse, ~10% of the k19 could be delivered into HEK-293T cells with an initial incubation concentration of 200 μM, as revealed by western blot analysis (Figure 1b). The concentration of ^15^N-labeled k19 in cells was only slightly deceased seven hours after electroporation (Figure 1b), indicating a long half-time of the protein in HEK-293T cells. Immunofluorescence imaging experiments further confirmed that the ^15^N-labeled k19 was successfully delivered into cells. Moreover, we found that k19 is mainly distributed in the cytoplasm, and it partially colocalizes with both MT and F-actin (Figure 1c). This result indicates that k19 may interact with different components in the cell, in contrast to another amyloid protein α-synuclein (α-syn), which has been previously studied by in-cell NMR and was found to be evenly distributed in the cytosol but did not exhibit specific interactions with other proteins [6]. 

To acquire a better quality and reproducible 2D NMR spectrum of the electroporated ^15^N-labeled k19 in HEK-293T cells, we optimized the Bruker standard SOFAST-HMQC pulse [40,41] with the delay time (D1) set to 0.29 s and the optimized ^1^H shape pulse. The 2D NMR spectrum was collected with 80 scans and 1024 × 128 complex points for ^1^H (14 ppm) and ^15^N (24 ppm), respectively, which resulted in a total of one hour for data collection. The 2D NMR spectra of k19 in HEK-293T cells with 3 and 7 h recovery were collected and displayed (Figure 1d). Western blot analysis confirmed that the collected NMR signals were mainly derived from the electroporated k19 in cells without significant leaking of k19 into the culture medium (Figure 1b). The NMR spectrum of k19 in cells shows similar patterns of crosspeaks to that of pure k19 in aqueous solution (Figure 1d). However, most resonances exhibited varied signal broadening and attenuation, especially the crosspeaks of residues 306VQIVYK311 (PHF6) that were previously identified to be crucial for fibrous Tau aggregation [42], exhibits significant broadening signals beyond detection (Figure 1e). This result suggests that k19 may interact with certain binding partners in cells, which is consistent with our imaging data that k19 colocalizes with MT and F-actin. Of note, two additional crosspeaks appeared in the in-cell NMR spectra; these two resonances exhibited enhanced signal intensities with the increasing recovery time without chemical shift perturbations, which may have been caused by a post-translational modification (e.g., phosphorylation, acetylation) on a particular residue of k19 in the HEK-293T cells. 

### 2.2. In Vitro NMR Characterization of Tau k19 with Crowding Agents

**Figure 2:**
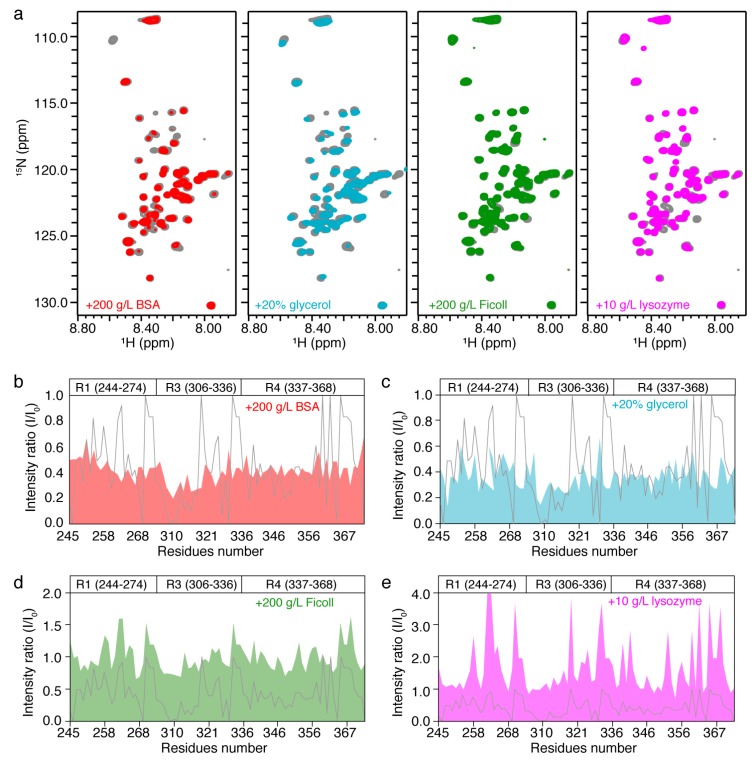
NMR characterization of k19 with crowding agents. (**a**) From left to right, overlay of the 2D ^1^H-^15^N HSQC spectra of 50 µM k19 in the absence (grey) and presence of 200 g/L BSA (red), 20% glycerol (blue), 200 g/L Ficoll (green), and 10 g/L lysozyme (magenta), respectively; (**b**–**e**) Residue-specific intensity ratio (I/I_0_) of k19 in the presence of 200 g/L BSA (**b**), 20% glycerol (**c**), 200 g/L Ficoll (**d**), and 10 g/L lysozyme (**e**) to k19 in buffer. The relative intensity ratio (I/I_0_) of k19 in HEK-293T cells to that of k19 in buffer is displayed as the grey line as a contrast.

We next asked whether the crowding effect in the intracellular environment contributes to the specific pattern of signal broadening and attenuation of k19 observed in the in-cell NMR spectrum. To mimic the intracellular crowding environment, we selected four different types of crowding agents including bovine serum albumin (BSA, 200 g/L), Ficoll (200 g/L), lysozyme (10 g/L) and glycerol (20%: *v*/*v*) and measured the HSQC spectra of purified ^15^N-labeled k19 in the presence of different agents, respectively. We found that addition of BSA and glycerol resulted in the global signal broadening of k19 (Figure 2a–c). On the contrary, addition with Ficoll and lysozyme caused significant enhancement of signal intensities for certain residues of k19, such as those around the sequence Lys-(Ile/Cys)-Gly-Ser (KXGS motif) of k19 (K257, S258, S262, T263, G323, S324, G326, and S352) and the subsequent sequence of Pro-Gly-Gly-Gly (PGGG motif, G271G272G273, G333G334G335, and G365G366G367N368), and moderate effects on the rest (Figure 2a,d,e). These results suggest that different crowding agents can influence the HSQC signal intensities of k19 in distinct ways. Whereas, none of these four crowding agents caused a similar intensity change profile as that of k19 in cells, indicating that the NMR spectrum of intracellular k19 may not be mainly due to crowding in the intracellular environment. 

### 2.3. In Vitro NMR Characterization of Tau k19 with MT and F-Actin

**Figure 3:**
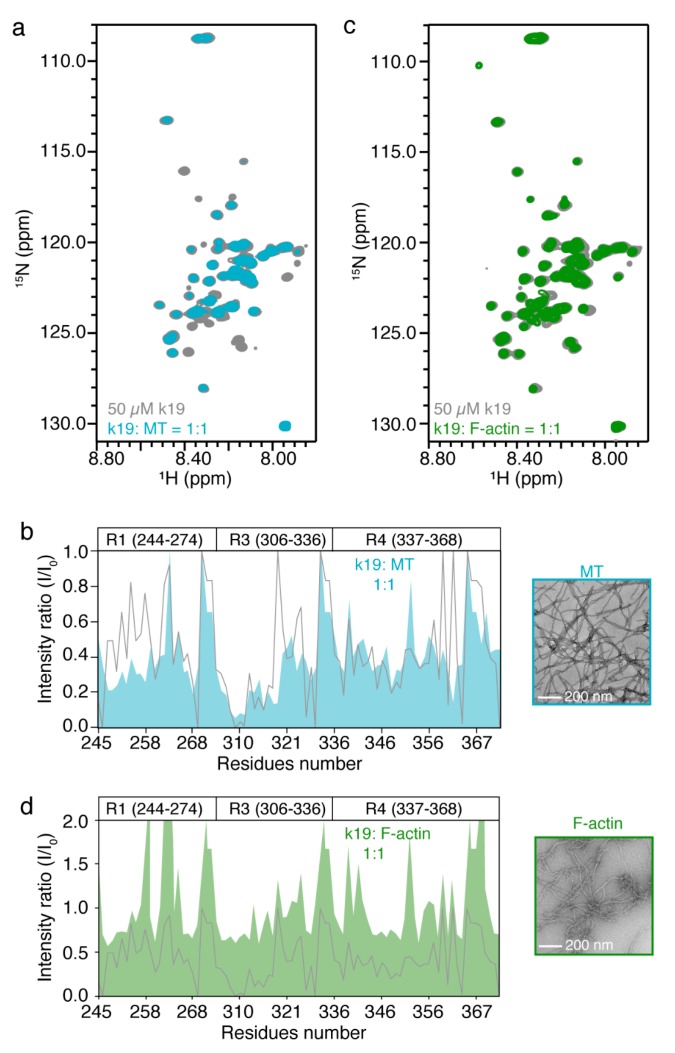
NMR characterization of k19 with microtubule (MT) and F-actin in vitro. (**a**,**c**) Overlay of the 2D ^1^H-^15^N HSQC spectra of 50 μM k19 in the absence (grey) or presence of MT (**a**, **blue**) and F-actin (**c**, **green**) at a molar ratio of 1:1, respectively; (**b**,**d**) Residue-specific intensity ratio (I/I_0_) of k19 in the presence of MT (**b**) and F-actin (**d**) to that of k19 in buffer, respectively. The relative intensity ratio (I/I_0_) of k19 in HEK-293T cells to that of k19 in buffer is displayed as a grey line as a contrast. TEM images of MT and F-actin used in the NMR sample are displayed on the right.

Tau protein was reported to bind and modulate the stability and dynamics of MT and F-actin, and can partially colocalize with both of them in cells based on our immunofluorescence imaging results. Thus, we asked how the binding of the MT and F-actin influence the characteristics of the k19 HSQC spectrum in vitro. We firstly prepared MT and F-actin from tubulin and actin, respectively, in vitro, and checked their formations by negative-staining transmission electron microscopy (TEM, Figure 3b,d). After centrifugation, the pellets of MT and F-actin were resuspended in NMR buffer and incubated with ^15^N-labeled k19, respectively. As shown in Figure 3a, the addition of MT caused severe signal broadening of k19. Notably, the resulting spectrum resembled the major characteristics of that of k19 acquired in HEK-293T cells, especially the PHF6 region, which exhibited significant broadening signals beyond detection. However, addition of MT resulted in a more severe signal intensity reduction of the N-terminal region of R1, which is the most dramatic difference between the two spectra (Figure 3b). In contrast, addition of F-actin results in the global decrease of NMR signals all over the k19 sequence. While, the residues around the KXGS (K257, S258, S262, T263, G323, G326, and S356) and PGGG (G271-G273, G333-G335, and G365-T369) motifs of k19 exhibit enhancement of the HSQC signal intensities (Figure 3c,d). Together, our results demonstrate that k19 can interact with both MT and F-actin in vitro through distinct binding interfaces. Moreover, the spectrum of k19 in complex with MT fits well with the in-cell NMR spectrum of k19, indicating that electroporated ^15^N-labeled k19 may predominantly bind to MT via its PHF6 region in HEK-293T cells.

### 2.4. In-Cell NMR of MARK2 Phosphorylated k19

**Figure 4:**
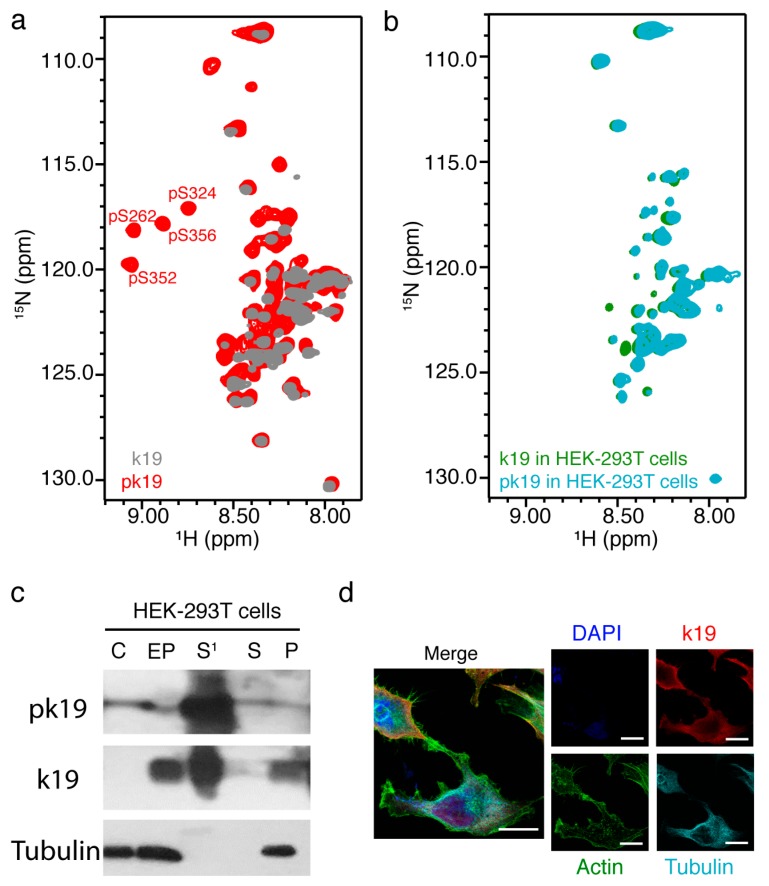
Characterization of the electroporated pk19 in mammalian cells. (**a**) Overlay of the 2D ^1^H-^15^N HSQC spectra of MARK2 phosphorylated k19 (pk19, red) and k19 (grey). The four phosphorylated serine residues including pS262, pS324, pS352, and pS356 are labeled. (**b**) Overlay of 2D in-cell NMR spectra of pk19 (blue) and k19 (green) in HEK-293T cells; (**c**) Western blot analysis of pk19 electroporated into HEK-293T cells. C, EP, S^1^, S, and P stand for untreated control cells, pk19 electroporated cells, supernatant medium of the cell–protein mixture after electroporation, supernatant medium and cell pellet of the in-cell NMR sample after NMR experiment collection, respectively. The antibody used for pk19 can only detect Tau when phosphorylated at S356, while the antibody for k19 can recognize both non-phosphorylated and phosphorylated S262 of Tau; (**d**) Immunofluorescence detection of delivered pk19 in SH-SY5Y cells after seven hours’ recovery. Scale bar, 7.5 μm. The antibody used here can recognize both non-phosphorylated and phosphorylated S262 of Tau.

Hyperphosphorylation of Tau by microtubule affinity regulating kinase 2 (MARK2) was reported as a key event in disrupting the MT binding of Tau and promoting its abnormal aggregation, which is closely associated with Alzheimer’s disease (AD) [35]. We next asked how the disease-associated MARK2-phosphorylated Tau (pk19) behaves in HEK-293T cells [33,35]. We first prepared the pk19 in vitro. Previous studies showed that MARK2 could phosphorylate the serine residues in the KXGS motifs of Tau’s repeat domain [43]. Consistently, we found that MARK2 can phosphorylate k19 at S262, S324, S352, and S356, as shown in the HSQC spectrum with the crosspeaks of these four residues featuring a significant downfield shift caused by phosphorylation (Figure 4a). We then electroporated the ^15^N-labeled pk19 into the HEK-293T cells and acquired the in-cell NMR spectrum. Intriguingly, the 2D NMR spectrum of pk19 in HEK-293T cells was the same as that of k19 in cells (Figure 4b). The phosphorylation of k19 on the four serine residues by MARK2 was eliminated once the pk19 protein was delivered into the cells. Western blot analysis further confirmed that only unphosphorylated k19 but not pk19 (anti-phosphorylated S356 Tau) could be detected in cells right after electroporation (Figure 4c). Moreover, after being dephosphorylated, delivered pk19 also distributed in the cytoplasm, and partially colocalized with both MT and F-actin (Figure 4d). Together, our data demonstrate that the disease-related pk19 was immediately dephosphorylated after being delivered into the HEK-293T cells, suggesting that the HEK-293T cells may utilize an unknown mechanism to protect Tau from being phosphorylated by MARK2 under normal conditions.

### 2.5. In-Cell NMR of Full-Length Tau40

**Figure 5:**
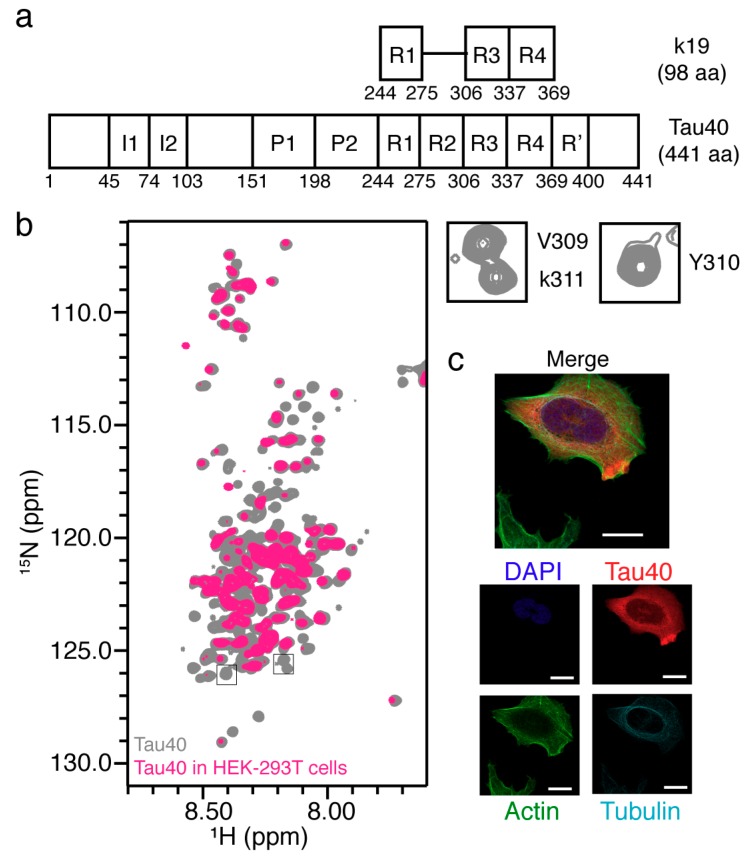
Characterization of the electroporated Tau40 in mammalian cells. (**a**) Bar diagram of full-length Tau40 and the fragment of k19. I1 and I2 stand for the two insertions in the N-terminal projection domain. The four MT-binding repeats are donated as R1-R4, while P1 and P2 represent the basic and proline-rich region preceding the repeats. R’ is the C-terminal flanking region; (**b**) Overlay of 2D in-cell NMR spectra of Tau40 in HEK-293T cells and in buffer (grey), the three crosspeaks of residues in the PHF6 region including V309, Y310, and Y311 are zoomed in on the right; (**c**) Immunofluorescence detection of delivered Tau40 in SH-SY5Y cells after seven hours’ recovery. Scale bar, 7.5 μm.

Finally, we sought to acquire the in-cell NMR spectrum of the largest isoform of Tau—Tau40 which contains 441 amino acids (Figure 5a). Firstly, we purified the ^15^N-labeled Tau40 in vitro and collected its 2D HSQC spectrum in aqueous solution (Figure 5b). The spectrum revealed a narrow and highly congested cluster of the amide proton signals, indicating that Tau40 adopts an intrinsically disordered conformation in an aqueous solution, which is consistent with previous studies [44]. Next, we electroporated the ^15^N-labeled Tau40 into the HEK-293T cells. The 2D in-cell NMR spectrum showed that a lot of resonances suffered severe signal broadening (Figure 5a), indicating that Tau40 binds to potential partners in cells. Moreover, similar to the in-cell NMR spectrum of k19, we found that most residues in the MT-binding repeats of Tau40 revealed significant signal intensity reduction, especially the residues around the PHF6 region. The signals of residues V309, Y310, and K311 in the in-cell NMR spectrum of Tau40 was completely unobservable, suggesting that these residues might bind to MT which is consistent with that of k19. Immunofluorescence imaging further showed that the delivered Tau40 was predominantly distributed in the cytoplasm and partially colocalized with MT and F-actin which is similar to electroporated k19 (Figure 5c). Taken together, our results reveal that Tau40 utilizes its MT-binding repeats to bind MT in mammalian cells.

## 3. Discussion

As introduced in 1975 [45], the in-cell NMR method has greatly expanded the toolbox of structural biology to explore the structure and dynamics of proteins in cells. Particularly, recent in-cell NMR studies on α-syn elegantly demonstrated that α-syn adopts as a monomer with a compacted disordered conformation in the cellular environment [6], and methionine-oxidized α-syn at the N-terminal, but not at the C-terminal, can be repaired by an intracellular reductase, revealing the general mechanism of site-selective α-syn repair in cells [21]. Herein, we utilized the in-cell NMR method to investigate the structure of the two isoforms of Tau, k19 and Tau40, in HEK-293T cells. We found that exogenously delivered k19 and Tau40 remain largely disordered in cells. Moreover, the MT-binding repeats of both two isoforms can bind to MT and F-actin in cells. Intriguingly, we found two additional unidentified crosspeaks for the delivered k19 in cells implying that post-translational modification may have occurred once k19 was delivered into the HEK-293T cells. However, the positions of these two crosspeaks in the NMR spectrum did not match with the phosphorylated sites studied previously by NMR in vitro [46,47,48,49]. A previous in-cell NMR study revealed that α-syn is acetylated at the N-terminal once electroporated into mammalian cells. Therefore, Tau might be acetylated or modified by other post-translational modifications such as methylation or phosphorylation in mammalian cells; this will be studied through combining protein mass spectrometry in the future. Notably, we observed that the exogenously delivered pk19, which is closely associated with disease, was immediately dephosphorylated in HEK-293T cells. This result implies that, in a normal cellular environment, the cell is engaged with a sophisticated post-translational modification system that safeguards the proper post-translational modification on different proteins and corrects abnormal modifications—in this case, MARK2-mediated phosphorylation on k19. Dysregulation of this system upon aging or under disease conditions may lead to hyperphosphorylation of Tau and consequently to abnormal aggregation.

We noticed that the overall 2D HSQC spectrum of electroporated Tau40 in the HEK-293T cells was similar to the previously acquired spectrum of Tau40 in the *Xenopus laevis* oocytes, delivered using microinjection [16]. However, we did not observe the additional resonances for Tau40 in HEK-293T cells which was previously identified as a possible phosphorylation resonance of Tau40 modified in *Xenopus laevis* oocytes. A recent in-cell NMR study revealed that cell type specifically contributes to the biological and pathological behaviors of α-syn in different intracellular environments [6,50]. Therefore, different cell types may feature distinct intracellular environment as well as distinct post-translational modification systems. Further in-cell NMR studies of Tau in neuron or neuronal-like cells may prove to be of great importance in revealing the physiological in-cell structure and dynamics of Tau.

## 4. Materials and Methods

### 4.1. Protein Overexpression and Purification

Human Tau40/k19 was expressed and purified on the basis of a previously described method [51]. Briefly, expression of Tau40/k19 was induced in *E. coli* BL21 (DE3) by addition of 0.5 mM isopropyl β-D-1-thiogalactopyranoside (IPTG) with the OD_600_ of 0.4–0.6 and grew overnight at 20 °C. After harvesting the cells, Tau40/k19 was purified by a HighTrap HP SP (5 mL) column (GE Healthcare, *Chicago*, IL, USA), followed by a Superdex 75 gel filtration column (GE Healthcare, *Chicago*, IL, USA). The final purified protein was buffer exchanged to NMR buffer (pH 6.5, 50 mM sodium phosphate buffer (PBS) and 50 mM NaCl), concentrated, and stored at −80 °C. Protein concentration was determined bybicinchoninic acid(BCA) assay (Thermo Fisher Scientific, Waltham, MA, USA).

For ^15^N- or ^15^N/^13^C-labeled proteins, protein expression and purification were the same as that for unlabeled proteins except that the cells were grown in M9 minimal medium with ^15^NH_4_Cl (1 g/L) in the absence or presence of ^13^C_6_-glucose (2 g/L).

### 4.2. In Vitro k19 Phosphorylation

Phosphorylation of k19 by MARK2 kinase was carried out following a method described previously [47]. Briefly, k19 was incubated with a hyperactive variant (T208E) of cat MARK2 [52] at a molar ratio of 10:1 in a buffer of 50 mM Hepes, pH 8.0, 150 mM KCl, 10 mM MgCl_2_, 5 mM ethylene glycol tetraacetic acid (EGTA), 1 mM phenylmethylsulfonyl fluoride (PMSF), 1 mM dithiothreitol (DTT), 2 mM ATP (Sigma, Saint Louis, MO, USA), and protease inhibitor cocktail (Roche, Basel, Swizerland) at 30 °C overnight. Phosphorylated protein was further purified by high-performance liquid chromatography (HPLC) (Agilent, Santa Clara, CA, USA) to remove kinase, and lyophilized. The sites and degrees of phosphorylation were quantified using 2D ^1^H-^15^N HSQC spectrum according to the previously publications [47,53].

### 4.3. Electroporation of Purified Proteins into Mammalian Cells

Human HEK-293T (ATCC, CRL-3216) and SH-SY5Y (ATCC, CRL-2266) cells were cultured following the methods provided by ATCC (Manassas, VA, USA). Both cell lines are routinely tested for mycoplasma contaminations and are mycoplasma free.

In-cell NMR samples were prepared using the modified protocol according to the previous publication [6]. The cells were passaged about 4–6 times prior to NMR experiments. The cells were collected by trypsinization and washed with PBS three times. Purified Tau/k19/pk19 (~1 mM) was diluted to a final concentration of 200 μM with Buffer R supplied in the Neon transfection system kit (Invitrogen, MPK10025, Carlsbad, CA, USA). Then cells in PBS were pellet again and then resuspended with the Tau/k19/pk19 solution to a cell density of ~8 × 10^7^ per mL. Electroporation was conducted using 100 μL of the cell-protein mixture with a pulse program of 1400 V (pulse voltage), 20 milliseconds (ms) (pulse width), and 1 pulse by the Neon transfection system (Invitrogen, MPK5000, Carlsbad, CA, USA).

For immunofluorescence and western blotting experiments, aliquots of 0.5 × 10^6^ cells were added to separate wells of a 24-well plate, filled with 0.5 mL media. For in-cell NMR samples of k19, aliquots of 4~8 × 10^6^ cells were added to eight 10-cm dishes with 10 mL media and cultured after 3~7 h for cell recovery. For pk19 and Tau40 samples, no recovery step was used. Then the cells were harvested and washed with PBS for four times. The in-cell NMR samples were taken up in pH-stable L-15 medium (Gibco, 11415064, Waltham, MA, USA) to a total volume of 500 μL with 10% D_2_O and settled into the NMR tube.

### 4.4. Western Blot

To determine the delivered protein in cells and check the leakage of the in-cell NMR samples, each cell sample was centrifuged at 300 *g* for 3 min immediately after the experiment. Then the supernatants and pellets were resuspended in Laemmli buffer to a final volume of 500 μL, and boiled for 10 min. These samples were diluted by 10 times, and loaded to a 15% SDS-PAGE gel. The primary antibodies used were listed as follows: Tau/k19 (abcam, ab64193, 1:1000), pk19 (abcam, ab75603, 1:1000) and tubulin (abcam, ab7291, 1:1000). Delivered k19 in HEK-293T cells was ~25 μM measured by ImageJ (Available online: https://imagej.net/Welcome) [54].

### 4.5. Immunostaining of Cultured Cells

For immunofluorescence imaging of control and electroporated cells as fixed specimens, cells were recovered at 37 °C for 7–8 h on poly-D-lysine-coated coverslips in 24-well plates. Cells were washed by pre-warmed PBS three times to remove extracellular proteins, then fixed in 4% (*w*/*v*) paraformaldehyde in PBS for 30 min and permeabilized with 0.1% (*v*/*v*) Triton X-100 in PBS for 20 min. After washing with PBS for three times, cells were blocked with 10% BSA in PBS for 1 h followed by incubation of antibodies at 4 °C overnight, including anti-Tau (abcam, ab64193, 1:1000), anti-alpha tubulin (abcam, ab7291, 1:1000), and fluorescein isothiocyanate-labeled phalloidin (Yeasen, 40736ES75, 1:200) for F-actin along with cell membrane skeleton. Slides were washed 3 × 10 min with PBS and nuclei stained by antifade mountant coupled DAPI (Invitrogen, P36935, Carlsbad, CA, USA). The samples were observed by a confocal microscope (Lecia, SP8, Wetzlar, Germany).

### 4.6. MT Preparation

Tubulin (catalog no. T240, Cytoskeleton) was incubated at concentrations higher than 200 μM in microtubule assembly buffer (100 mM Pipes, pH 6.9, 1 mM EDTA, 1 mM MgSO_4_, 1 mM DTT) in the presence of 1 mM GTP at 37 °C for 5 min. After addition of 100 μM paclitaxel (Sigma, Saint Louis, MO, USA), the polymerization was performed for 60 min at 37 °C. The integrity of MTs was checked by negative- staining TEM. Just before NMR experiments, samples were centrifuged at 14,000 rpm for 15 min, then the pellets were resuspended in NMR buffer.

### 4.7. F-Actin Preparation

Non-muscle human actin (catalog no. APHL99, Cytoskeleton) was first dissolved by 100 μL water to reach a concentration of 10 mg/mL and then diluted to 1 mg/mL with the actin buffer (5 mM Tris-HCl, pH 8.0, 0.2 mM CaCl_2_, 0.2 mM ATP, 0.5 mM DTT, 5% (*w*/*v*) sucrose and 1% (*w*/*v*) dextran). After centrifugation at 14,000 rpm for 15 min, the supernatant was kept. Then actin was polymerized by addition of polymerization buffer (1/10th the volume; 100 mM Tris-HCl, pH 7.5, 20 mM MgCl_2_, 500 mM KCl and 10 mM ATP) at room temperature for 1 h. The integrity of F-actins was checked by negative-staining TEM. Just before the NMR experiments, samples were spun down at 14,000 rpm for 15 min, then the pellets were resuspended in NMR buffer.

### 4.8. Transmission Electron Microscopy (TEM)

TEM images were collected on a Tecnai G2 Spirit TEM operated at an accelerating voltage of 120 kV. Samples (8 μL) were deposited on carbon-coated grids for 45 s. The grids were then washed twice with ddH_2_O (8 μL) and incubated with 8 μL uranyl acetate (2%, *v*/*v*) for staining. Images were recorded using a 4K × 4K charge-coupled device camera (BM-Eagle, FEI Tecnai, Waltham, MA, USA).

### 4.9. In-Cell and In Vitro NMR Spectroscopy

All the NMR experiments were carried out at 25 °C on a Bruker 900 or 600 MHz spectrometer equipped with a cryogenic probe. The buffer used in all in vitro NMR assays was 50 mM sodium phosphate buffer (pH 6.5) containing 50 mM NaCl and 10% D_2_O (*v*/*v*). Backbone resonance assignment of k19 and pk19 was accomplished based on the collected 3D HNCACB and CBCACONH spectra and assignments from previous studies [47,53].

For the in-cell NMR samples, Bruker standard SOFAST-HMQC pulse sequence [40,41] was used with the delay time (D1) of 0.29 s and the ^1^H shape pulse efficient was optimized for collecting the 2D NMR spectrum. 1024 × 128 complex points were used for ^1^H (14 ppm) and ^15^N (24 ppm), respectively. The 2D SOFAST-HMQC spectrum was collected with 80 scans resulting in totally one hour experiment time. To calculate the residue-resolved relative intensity ratio (Y) of k19 in cells (I) versus in buffer (I_0_) in Figure 1e, the equation Y = [I(X)/I_0_(X)]/[I(333)/I_0_(333)] was used for each residue X, where the intensity ratio of residue X was normalized by that of the highly flexible residue G333.

For in vitro titration assays, each NMR sample was freshly prepared from high concentration protein stocks with a total volume of 500 µL (10% D_2_O). Each 2D ^1^H-^15^N HSQC spectrum was collected with 16 scans per transient and complex points of 2048 × 160 for ^1^H (14 ppm) and ^15^N (24 ppm), respectively. All NMR data were processed by NMRpipe [55] and analyzed by NMRViewJ [56].
